# The Impact of Opening Wedge High Tibial Osteotomy on Spinal Alignment

**DOI:** 10.7759/cureus.89173

**Published:** 2025-07-31

**Authors:** Tetsuro Shimada, Akira Maeyama, Tetsuro Ishimatsu, Taiki Matsunaga, Kotaro Miyazaki, Takuaki Yamamoto

**Affiliations:** 1 Department of Orthopaedic Surgery, Fukuoka University Faculty of Medicine, Fukuoka, JPN

**Keywords:** lumbopelvic alignment, owhto, pelvic tilt, pi–ll mismatch, spinopelvic sagittal alignment, whole-spine radiographs

## Abstract

Introduction

Flexion contracture of the knee has been reported to induce forward trunk inclination and pelvic retroversion, whereas the progression of pelvic retroversion may further exacerbate knee joint symptoms, suggesting a close relationship between the knee and spinal alignment. The purpose of this study was to investigate the effects of lower limb alignment changes after opening wedge high tibial osteotomy (OWHTO) on spinopelvic sagittal alignment.

Methods

We retrospectively analyzed 34 knees that underwent OWHTO for medial compartment knee osteoarthritis between 2023 and 2025. Standing full-length lower limb and whole-spine radiographs were obtained preoperatively and at one year postoperatively. Lower limb alignment parameters, i.e., the percentage of mechanical axis (MA), mechanical medial proximal tibial angle (mMPTA), mechanical lateral distal femoral angle (mLDFA), joint line convergence angle (JLCA), and posterior tibial slope (PTS), were evaluated. Spinopelvic parameters included pelvic tilt (PT), sacral slope (SS), pelvic incidence (PI), thoracic kyphosis (TK), lumbar lordosis (LL), and the mismatch between PI and LL (PI-LL) Clinical outcomes were assessed using the Knee Injury and Osteoarthritis Outcome Score (KOOS) before surgery and at one year postoperatively. Correlations between postoperative spinopelvic parameters and clinical outcomes were also examined.

Results

Pelvic tilt and the mismatch between PI and LL significantly decreased following OWHTO (p = 0.003 and p = 0.044, respectively), indicating improvement in spinopelvic sagittal alignment. However, no significant correlations were found between changes in spinal alignment and postoperative scores on the KOOS.

Conclusion

Both pelvic tilt and the mismatch between PI and LL, which reflect lumbopelvic alignment, were significantly decreased after OWHTO. This may have arisen because the correction of knee alignment improved the load distribution in the lower limbs, which in turn contributed to the improvement of lumbopelvic alignment. Our findings suggest that OWHTO not only corrects lower limb alignment but may also have a positive effect on lumbopelvic alignment.

## Introduction

Surgical treatments such as around-knee osteotomy (AKO) and total knee arthroplasty have shown favorable long-term outcomes in patients with advanced medial compartment knee osteoarthritis (OA) [1]. Total knee arthroplasty is indicated for patients with moderate-to-severe knee OA, whereas AKO is typically selected for relatively younger and more active patients [2-5]. Around-knee osteotomy aims to correct lower limb alignment, offload the medial compartment of the knee, and restore joint function. Several surgical techniques have been developed for AKO, including opening wedge high tibial osteotomy (OWHTO), closed wedge high tibial osteotomy, and double-level osteotomy [6,7]. Among these, OWHTO is a relatively simple procedure, and favorable long-term clinical outcomes have been reported [8-10]. However, the prognostic factors associated with clinical outcomes after OWHTO remain controversial, with studies citing variables such as age, body mass index, and the severity of OA [11-13]. Furthermore, the secondary effects on adjacent joints, such as the hip and ankle, resulting from lower limb realignment after OWHTO have not yet been fully elucidated. The potential effects of OWHTO on spinal alignment also remain unclear.

Spinal deformities are known to progress with aging and influence the knee joint. In the sagittal plane, spinal alignment is characterized by a decrease in lumbar lordosis (LL) and an increase in pelvic retroversion [14]. A decrease in LL leads to pelvic retroversion, hip extension, knee flexion, and ankle dorsiflexion [15]. Moreover, pelvic retroversion caused by age-related decline in trunk function and spinal kyphosis has been reported to potentially contribute to the development of knee OA through disruption of the kinetic chain [16]. Pelvic retroversion has been suggested to generate a flexion moment at the knee joint, thereby accelerating knee flexion contracture and varus deformity, and potentially contributing to the progression of knee OA. Conversely, when knee flexion contracture occurs, it has been shown to affect the spine by reducing LL, which in turn leads to forward trunk inclination and anterior displacement of the C7 plumb line [17]. Otsuki et al. reported that pelvic retroversion progressively increases over time following OWHTO, leading to knee flexion contracture and deterioration of clinical outcomes in the knee joint [11]. However, their study evaluated pelvic tilt (PT) using the 'tilt ratio' measured on anteroposterior pelvic radiographs, which did not comprehensively capture overall spinal or sagittal alignment. To date, there have been no detailed reports examining whole-spine alignment, including sagittal parameters, in relation to morphological changes after OWHTO.

The purpose of this study was to investigate the effects of lower limb alignment changes after OWHTO on spinopelvic sagittal alignment, as assessed by standing lateral whole-spine radiographs. We hypothesized that realignment of the lower limb would influence spinopelvic parameters on lateral standing radiographs.

## Materials and methods

Patient selection

This retrospective, non-randomized, and sequential review study was approved by the Institutional Review Board of Fukuoka University Hospital (approval no. U23-07-014), and informed consent was obtained from all patients. Patients who underwent OWHTO for medial compartment knee OA at our institution between January 2023 and April 2025 were included in the study. All procedures were performed by three senior orthopedic surgeons.

**Figure 1:**
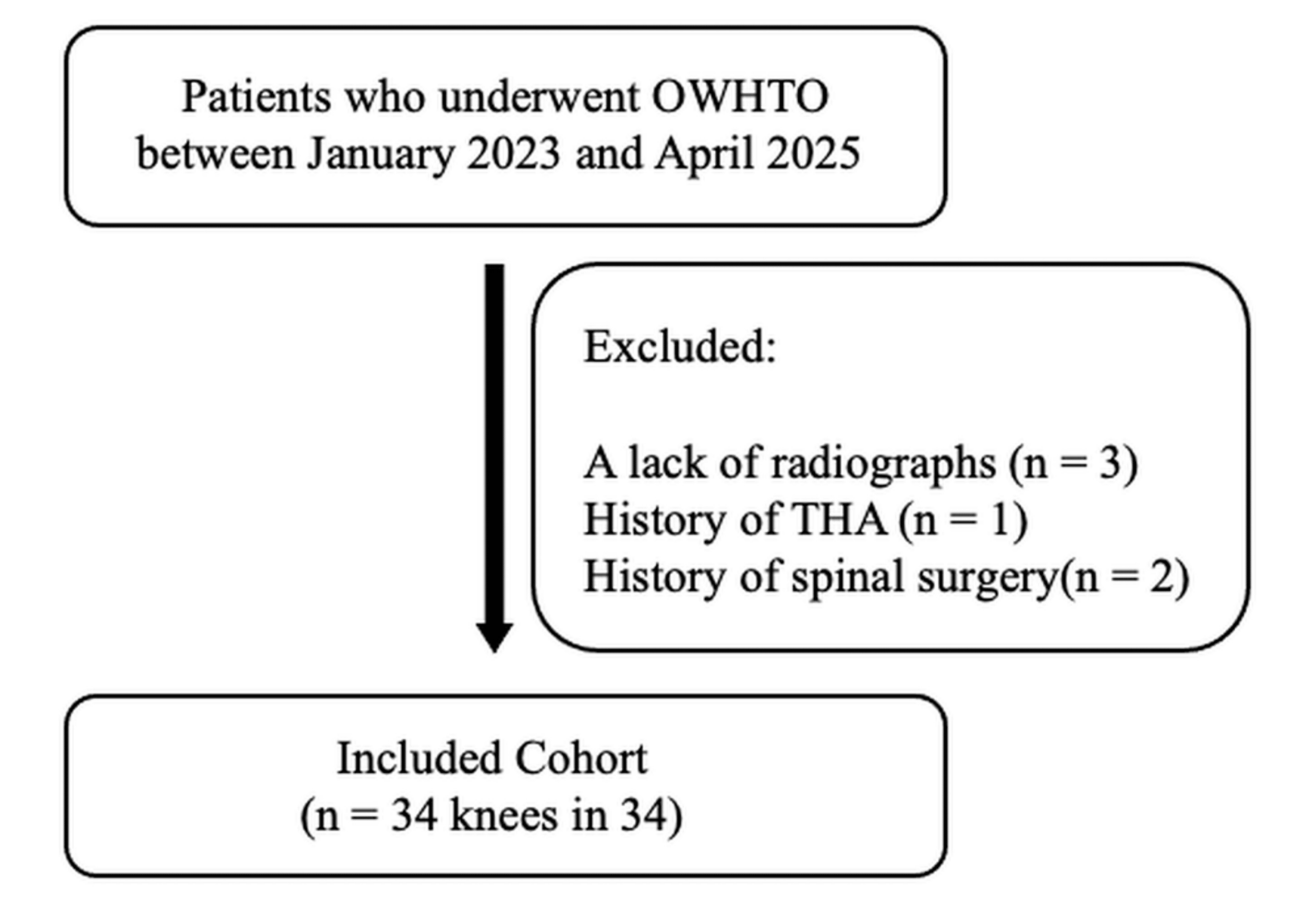
Study flowchart OWHTO: Opening wedge high tibial osteotomy; THA: Total hip arthroplasty

The surgical indications for OWHTO were as follows: (1) radiographic evidence of OA confined to the medial compartment of the knee, (2) primary OA (excluding inflammatory arthritis), (3) failure of conservative treatment, and (4) physically active patients capable of adhering to postoperative rehabilitation protocols. The exclusion criteria for OWHTO were as follows: (1) patients for whom whole-spine anteroposterior and lateral radiographs, as well as full-length weight-bearing anteroposterior and lateral radiographs of the lower extremity, were not available both preoperatively and one year postoperatively; (2) patients who had undergone total hip arthroplasty; (3) patients with a history of spinal surgery; (4) patients with pre-existing, symptomatic spinal conditions (e.g., degenerative spondylolisthesis, scoliosis) without a history of surgery; and (5) patients with neuromuscular disorders affecting posture. Ultimately, 34 knees of 34 patients were included in the study (Figure 1). No patients were lost to follow-up. The patient characteristics are summarized in Table 1.

**Table 1:** Demographic characteristics of patients Data are presented as mean ± standard deviation or number of patients (total n =34).

Characteristics	Values
Age (years)	62.2 ± 7.7 (range 36-75)
Gender (male/female)	9/25
Body mass index (kg/m^2^)	25.7 ± 4.0 (range 20.5-38.5)
Kellgren-Lawrence grade (I/II/III/IV)	0/12/19/3

Surgical procedure and postoperative rehabilitation

**Figure 2:**
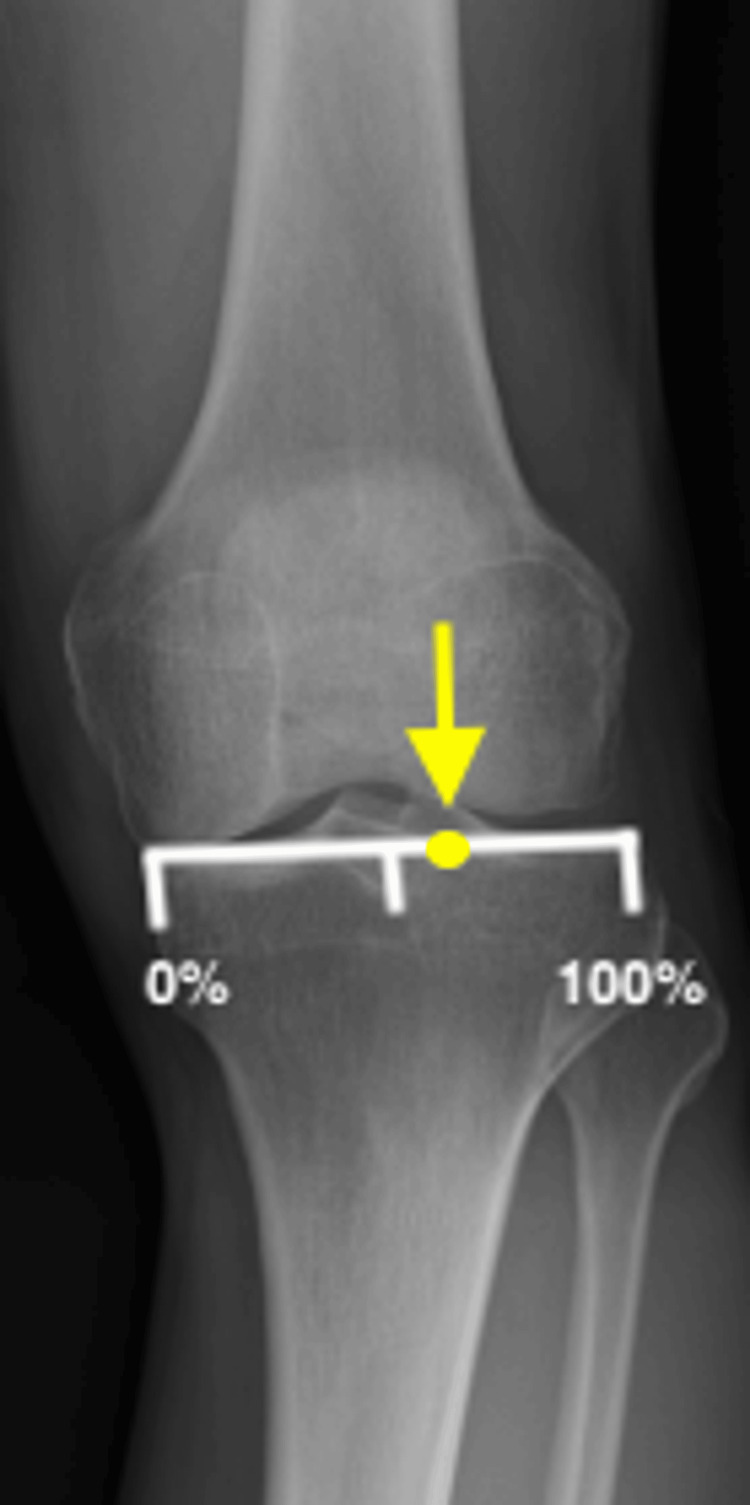
Fujisawa point (yellow arrow) located at 62.5% of the tibial plateau width from the medial edge.

During the preoperative planning, the target weight-bearing line was set at the Fujisawa point, located at 62.5% of the tibial plateau width from the medial edge, on standing full-length lower extremity radiographs [18] (Figure 2). Arthroscopy was routinely performed before OWHTO to evaluate the articular cartilage and meniscus. For cartilage lesions classified as grade 3 or higher according to the International Cartilage Repair Society classification, microfracture or drilling was performed. In cases of meniscal tears, partial meniscectomy or meniscal repair was performed.

A biplanar OWHTO was subsequently performed [19]. The osteotomy site was gradually opened using a spreader until the preoperatively planned mechanical medial proximal tibial angle (mMPTA) was achieved. The mMPTA was confirmed intraoperatively using a transparent acrylic angle gauge extending from the knee to the ankle joint. The gap width between the posteromedial cortices was measured. Two blocks of β-tricalcium phosphate with 60% porosity were inserted on the cortical bone side, and β-tricalcium phosphate granules with 75% porosity were used to fill the cancellous bone side. After repairing the pes anserinus, the medial osteotomy site was fixed using a locking plate (Tris Plate® from Olympus Terumo Biomaterials, Tokyo, JPN; or Anatomically Contoured Plate® from Zimmer Biomet, Warsaw, IN, USA).

Isometric quadriceps exercises, active ankle exercises, and straight leg raises were started on postoperative day three; partial (50%) weight-bearing was started at two weeks postoperatively; and full weight-bearing was permitted after four weeks postoperatively. However, in cases where microfracture was performed, partial (50%) weight-bearing was started at three weeks postoperatively, and full weight-bearing was permitted after five weeks postoperatively.

Radiographic evaluation

**Figure 3:**
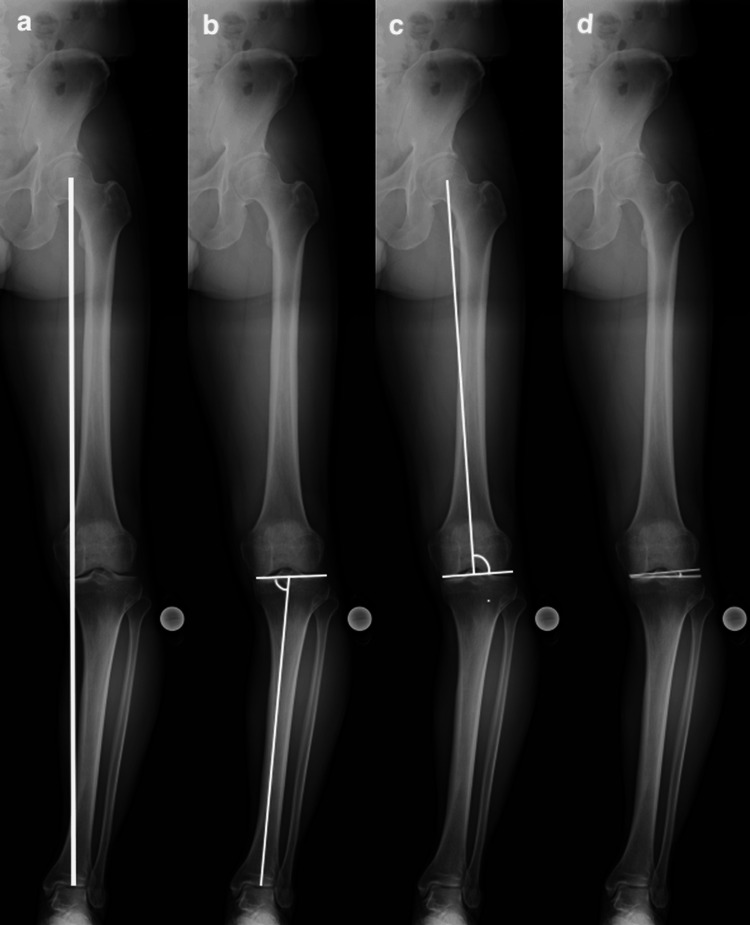
Radiographic parameters on single-leg standing whole-leg radiographs (a) MA: A straight line drawn from the center of the femoral head to the center of the ankle joint. The percentage of the MA is calculated as the ratio of the horizontal distance from the medial edge of the tibial plateau to the intersection of the MA. (b) mMPTA: The medial angle between the tibial MA and the proximal articular surface of the tibia. (c) mLDFA: The lateral angle between the femoral MA and the distal articular surface of the femur. (d) JLCA: The angle between the distal femoral articular surface and the proximal tibial articular surface. MA: Mechanical axis; mMPTA: Mechanical medial proximal tibial angle; mLDFA: Mechanical lateral distal femoral angle; JLCA: Joint line convergence angle Image credit: Author Tetsuro Shimada

**Figure 4:**
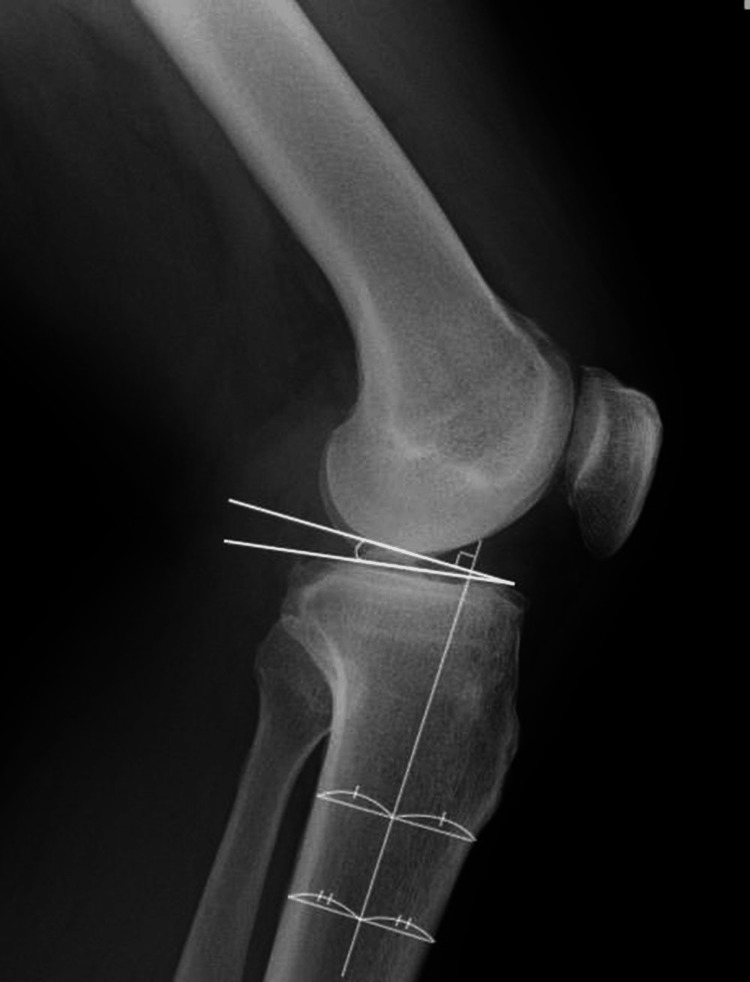
Lateral radiograph of the knee Seen is the PTS, the angle between the averaged slope of the medial and lateral tibial plateaus and a line representing the tibial diaphyseal axis. PTS: Posterior tibial slope Image credit: Author Tetsuro Shimada

Radiographic assessments were performed preoperatively and at one year postoperatively. The percentage of mechanical axis (MA), mMPTA, mechanical lateral distal femoral angle (MLDA), and joint line convergence angle (JLCA) were measured on standing full-length anteroposterior radiographs of the lower limb that were taken in a double-leg stance (Figure 3). The posterior tibial slope (PTS) was measured on the lateral radiographs of the knee (Figure 4).

**Figure 5:**
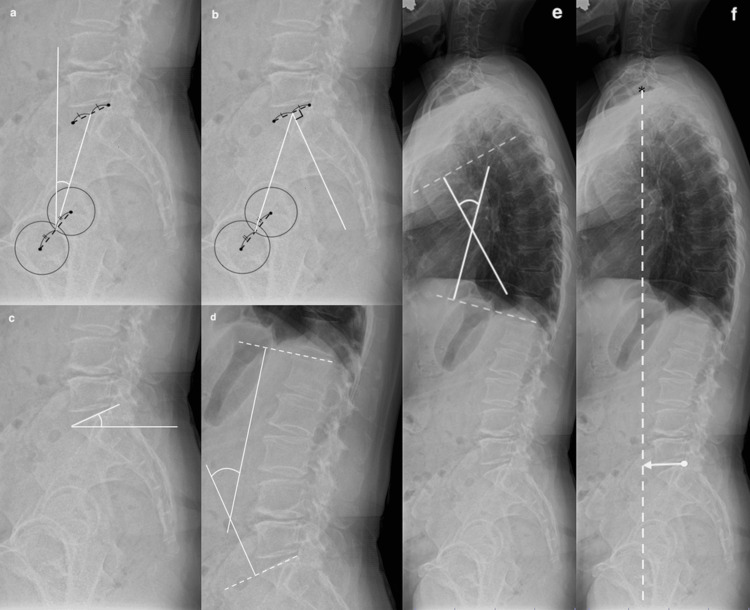
Radiographic parameters on whole-spine standing lateral radiographs (a) PT: The angle between the line from the center of the femoral heads to the midpoint of the S1 endplate and the vertical line. (b) PI: The angle between the line connecting the midpoint of the S1 endplate to the center of the femoral heads and the lines perpendicular to the S1 endplate. (c) SS: The angle between the superior endplate of S1 and the horizontal line. (d) LL: The angle between the lines perpendicular to the superior endplates of L1 and S1. (e) TK: The angle between the lines perpendicular to the superior endplate of T4 and the inferior endplate of T12. (f) SVA: The horizontal distance between the vertical line from the center of C7 and the posterosuperior corner of the S1 endplate (the asterisk indicates the center of the seventh cervical vertebra). PT: Pelvic tilt, PI: Pelvic incidence, SS: Sacral slope, LL: Lumbar lordosis, TK: Thoracic kyphosis, SVA: Sagittal vertical axis Image credit: Author Tetsuro Shimada

On standing lateral whole-spine radiographs taken in a double-leg stance, the following sagittal alignment parameters were measured: pelvic tilt (PT), pelvic incidence (PI), sacral slope (SS), lumbar lordosis (LL), thoracic kyphosis (TK), the sagittal vertical axis (SVA), and the mismatch between PI and LL (PI-LL) (Figure 5). The PT was measured on standing lateral whole-spine radiographs as the angle between the line from the center of the femoral heads to the midpoint of the S1 endplate and the vertical line. This differs from pelvic inclination, which is sometimes measured on anteroposterior pelvic radiographs. Sagittal spinal deformity is generally calculated as the difference between PI and LL [20-23]. The 'PI-LL' is the numerical difference between PI and LL and was used as an indicator of sagittal balance. The PI represents the anatomical relationship between the sacrum and the hip joints and reflects the morphology of the pelvis [23]. It is a fixed anatomical parameter that remains unaffected by aging or postural changes [23]. In contrast, LL is a dynamic lordotic angle that varies depending on posture and age [14]. A larger PI indicates a more retroverted pelvis, and individuals with a high PI require a greater degree of LL to maintain proper sagittal balance [20]. Both PI and LL are interrelated parameters that influence each other [20]. Clinical outcomes were evaluated pre- and postoperatively using the Knee Injury and Osteoarthritis Outcome Score (KOOS) [24].

Statistical analysis

Paired t-tests were used to evaluate pre- and postoperative differences in all measured parameters and KOOS. Spearman’s rank correlation coefficient analysis was performed to assess the relationships between KOOS subscales and each spinal alignment parameter. A p-value < 0.05 was considered statistically significant. Post hoc power analyses showed that a sample size of 27 achieved a power of 80% and a significance level of 5%, which verified the adequacy of our sample size for this analysis.

All measurements were performed independently by two senior orthopedic surgeons. The reliability of the measurements was assessed using intraclass correlation coefficients for both intra- and interobserver reliability. The intraclass correlation coefficients ranged from 0.81 to 1.00 for both intra- and interobserver variance. Statistical analyses were performed using SPSS Statistics for Windows version 23.0 (IBM Corp., Armonk, NY, USA). 

## Results

The radiographic parameters are presented in Tables 2-3. Compared with preoperative values, the mean postoperative percentage of mechanical axis was significantly improved (p < 0.001). The mMPTA and posterior tibial slope were significantly increased postoperatively (p < 0.001), whereas the joint line convergence angle was significantly decreased postoperatively (p < 0.001) (Table 2). With respect to spinopelvic sagittal alignment, both PT and PI-LL were significantly reduced postoperatively compared with the preoperative values (p = 0.003 and p = 0.029, respectively) (Table 3).

**Table 2:** Preoperative and postoperative radiographic measurements Data are presented as mean ± standard deviation. OWHTO: Opening wedge high tibial osteotomy; ROM: Range of motion; MA: Mechanical axis; mMPTA: Mechanical medial proximal tibial angle; mLDFA: Mechanical lateral distal femoral angle; JLCA: Joint line convergence angle; PTS: Posterior tibial slope

Parameter	Pre-OWHTO	Post-OWHTO	p-value
Flexion ROM (°)	133.6 ± 9.7	133.4 ± 9.2	0.940
Extension ROM (°)	-1.0 ± 2.0	-0.7 ± 1.9	0.503
MA (%)	27.1 ± 9.8	58.9 ± 8.3	< 0.001
mMPTA (°)	85.8 ± 1.7	91.8 ± 2.4	< 0.001
mLDFA (°)	88.5 ± 2.2	87.9 ± 1.9	0.088
JLCA (°)	2.4 ± 1.6	1.5 ± 1.4	< 0.001
PTS (°)	7.5 ± 2.4	10.3 ± 1.9	< 0.001

**Table 3:** Pre- and postoperative standing whole-spine radiographic measurements PT: Pelvic tilt; PI: Pelvic incidence; SS: Sacral slope; LL: Lumbar lordosis; TK: Thoracic kyphosis; SVA: Sagittal vertical axis; PI−LL: The mismatch between PI and LL; OWHTO: Opening wedge high tibial osteotomy

Parameter	Pre-OWHTO	Post-OWHTO	p-value
PT (°)	17.8 ± 8.8	14.2 ± 7.6	0.003
PI (°)	51.3 ± 10.7	51.1 ± 9.5	0.653
SS (°)	34.0 ± 8.9	36.5 ± 6.9	0.204
LL (°)	44.1 ± 10.9	46.0 ± 9.4	0.162
TK (°)	30.3 ± 9.1	30.0 ± 9.3	0.758
PI−LL (°)	7.6 ± 12.1	4.8 ± 9.7	0.029
SVA (mm)	32.0 ± 30.8	28.3 ± 25.2	0.885

All preoperative mean KOOS categories (total KOOS, pain, symptoms, activities of daily living, sports/recreation function, and knee-related quality of life) improved significantly postoperatively (Table 4). However, no significant correlations were found between postoperative KOOS categories and any of the spinopelvic alignment parameters.

**Table 4:** Preoperative and postoperative scores on the KOOS KOOS: Knee Injury Osteoarthritis Outcome Score; OWHTO: Opening wedge high tibial osteotomy

KOOS	Pre-OWHTO	Post-OWHTO	p-value
Total	43.5 ± 14.6	71.0 ± 19.5	<0.001
Symptoms	52.9 ± 20.6	82.2 ± 18.2	<0.001
Pain	48.9 ± 19.1	78.7 ± 18.5	<0.001
Activities of daily living	65.0 ± 16.6	83.4 ± 14.3	<0.001
Sports/Recreation	24.1 ± 16.2	52.8 ± 20.3	<0.001
Knee-related quality of life	26.8 ± 15.3	58.1 ± 16.7	<0.001

## Discussion

The most important finding of the present study is that PT and PI-LL were significantly reduced following OWHTO. This result suggests that OWHTO achieves not only mechanical correction of the lower limb axis but also potential improvements in lumbopelvic sagittal alignment.

Murata et al. [25] suggested that knee flexion contracture may contribute to pelvic retroversion and a decrease in LL, and proposed the concept of “knee-spine syndrome.” Yasuda et al. [22] found that in volunteers aged 50 years and older, the severity of knee OA was associated with increased pelvic retroversion and PI-LL mismatch. These changes were particularly associated with low back pain in female participants. These studies suggest that changes such as lower limb malalignment and restricted knee joint range of motion may affect lumbopelvic alignment. 

The OWHTO is an effective treatment for medial compartment knee OA, as it reduces knee flexion contracture and pain by shifting the weight-bearing axis laterally [19,26]. In this study, both PT and PI-LL improved despite no change in knee extension angle. A decrease in PT indicates an improvement from pelvic retroversion toward anterior PT, whereas a decrease in PI-LL reflects the correction of lumbopelvic sagittal alignment [20]. Kim et al. evaluated spinal alignment during gait before and after OWHTO using a 3D motion analysis system and reported significant improvements in all spinal alignment parameters postoperatively compared with the preoperative values [27]. Their study demonstrated that both forward trunk inclination and pelvic retroversion during gait were significantly improved after surgery. Although their study assessed postural changes during gait, this study is the first to quantitatively demonstrate improvements in sagittal alignment parameters, such as PT and PI-LL, using static standing whole-spine radiographs.

**Figure 6:**
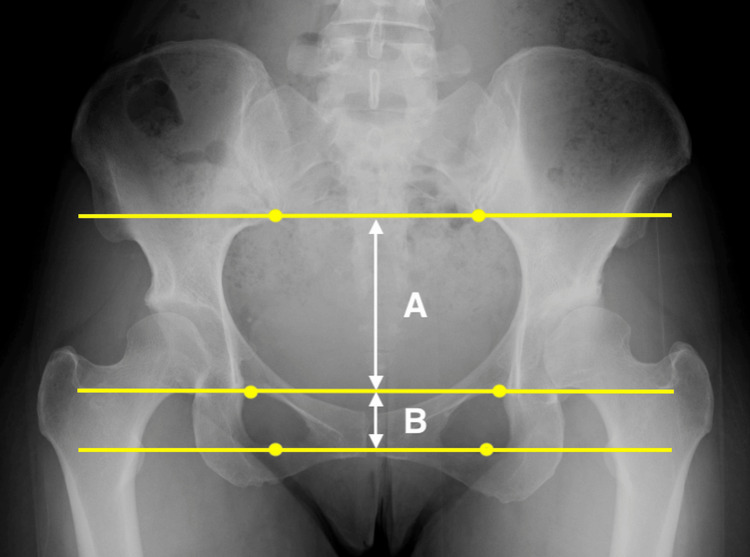
Assessment of the tilt ratio (B/A) Bilateral identification of the lower margin of the sacroiliac joint and the upper and lower borders of the obturator foramen is required to assess the tilt ratio.

In contrast, Otsuki et al. found no significant difference in pelvic inclination after OWHTO compared with preoperative measurements [11]. Their evaluation was based on the 'tilt ratio' proposed by Schwarz et al. [28], which differs from the measurement method used in the present study (Figure 6). This method has not been confirmed to correlate with the actual pelvic inclination on lateral radiographs. The differences in measurement methods may explain the discrepant findings. In this study, despite improvements in sagittal spinal alignment parameters following OWHTO, no significant correlations were found between changes in spinal alignment and clinical outcomes, as assessed by KOOS. However, KOOS is a knee-specific score that does not measure spine-related symptoms. Therefore, the absence of any correlations should be interpreted carefully.

The findings suggest that radiographic improvements in spinal alignment may not directly translate to improvements in patient-reported outcomes related to the knee. Future studies should include spine-specific clinical scores to better understand the clinical effects of lumbopelvic alignment changes. Otsuki et al. [11] reported that progression of pelvic retroversion after OWHTO correlates with knee flexion contracture in older patients and negatively impacts long-term clinical outcomes. The difference in the findings may be due to variations in the measurement methods and also differences in the observation periods. While the study by Otsuki et al. was conducted with long-term follow-up averaging 42.9 months postoperatively, the present study evaluated the outcomes at only one year postoperatively. The progression of pelvic retroversion and its impact on clinical symptoms may become more apparent over a longer period, and such changes may not have been fully captured within the observation period of the present study.

These findings also suggest that pelvic retroversion may adversely affect knee extension function and related symptoms. Pelvic retroversion is also known to increase the mechanical load in relation to lumbar alignment and elevate the risk of low back pain. Kitagawa et al. [29] reported that excessive pelvic retroversion is associated with increased PI-LL and low back pain originating from the lumbar spine. Based on these findings, pelvic retroversion may be detrimental for both the knee joint and the lumbar spine.

In summary, OWHTO may have a positive effect on pelvic and spinal alignment. However, these improvements do not appear to directly correlate with patients’ subjective knee symptoms or daily functional outcomes. Thus, the improvement in spinal alignment may serve only a supplementary role in the overall therapeutic effect of OWHTO.

The present study has several limitations. First, the study had a retrospective design, which may have introduced selection bias. Second, clinical evaluations of the lumbar spine, such as use of a visual analog scale or the Oswestry Disability Index [30], were not performed. Therefore, we were unable to evaluate whether the improvements in lumbopelvic alignment were associated with improvements in clinical symptoms such as low back pain. Future prospective studies incorporating these clinical measures are needed to clarify the relationships between the radiographic findings and patient-reported outcomes. Third, the sample size was relatively small, which may limit the generalizability of the findings. However, the number of patients exceeded the minimum required to achieve a statistical power of 0.8 in the post hoc analysis. Fourth, the imaging assessment in the study was based on radiographs only, and no detailed analyses using CT or other advanced imaging methods were performed. Incorporation of CT-based evaluations and collaboration with radiologists could enable more precise measurements, as these are considered important for future research.

## Conclusions

This study demonstrated significant improvements in PT and the PI-LL following OWHTO. These findings may indicate the restoration of lumbopelvic sagittal alignment after surgery. The results suggest that OWHTO is not only effective in mechanically correcting lower limb alignment but may also have a favorable influence on lumbopelvic alignment. Although these changes may help to improve posture, further studies are needed to clarify their clinical relevance, such as their effects on low back pain or spinal function.
